# Related Markers for the Precision Diagnosis of Complex Appendicitis in Children

**DOI:** 10.3389/fphar.2022.865303

**Published:** 2022-03-31

**Authors:** Jialin Zhou, Wenjing Xu, Jitao Wang, Zhe Fan

**Affiliations:** ^1^ Department of General Surgery, The Third People’s Hospital of Dalian, Dalian Medical University, Dalian, China; ^2^ Department of Central Laboratory, The Third People’s Hospital of Dalian, Dalian Medical University, Dalian, China; ^3^ Department of General Surgery, Zhongda Hospital, School of Medicine, Southeast University, Nanjing, China; ^4^ Department of Hepatobiliary Surgery, Xingtai People’s Hospital, Xingtai, China

**Keywords:** complex appendicitis, children, biomarkers, rating related, review

## Abstract

Acute appendicitis is the most common surgical emergency in children. Despite the high incidence rate of appendicitis, it is sometimes misdiagnosed or missed. Complex appendicitis (CA) in children is characterized by a critical condition, several complications, and high mortality. Precision distinguishing between simple appendicitis and CA correctly is key to choosing appropriate treatment. A safe, cheap, rapid, extensive and accurate diagnostic marker of appendicitis will be of great significance for emergency general surgeons to treat suspected CA. Many studies have investigated possible diagnostic markers for the diagnosis of CA in children. In this study, studies related to CA in children in recent years are summarized, and the related markers and scoring system for the diagnosis of CA in children are summarized.

## Background

**FIGURE 1:**
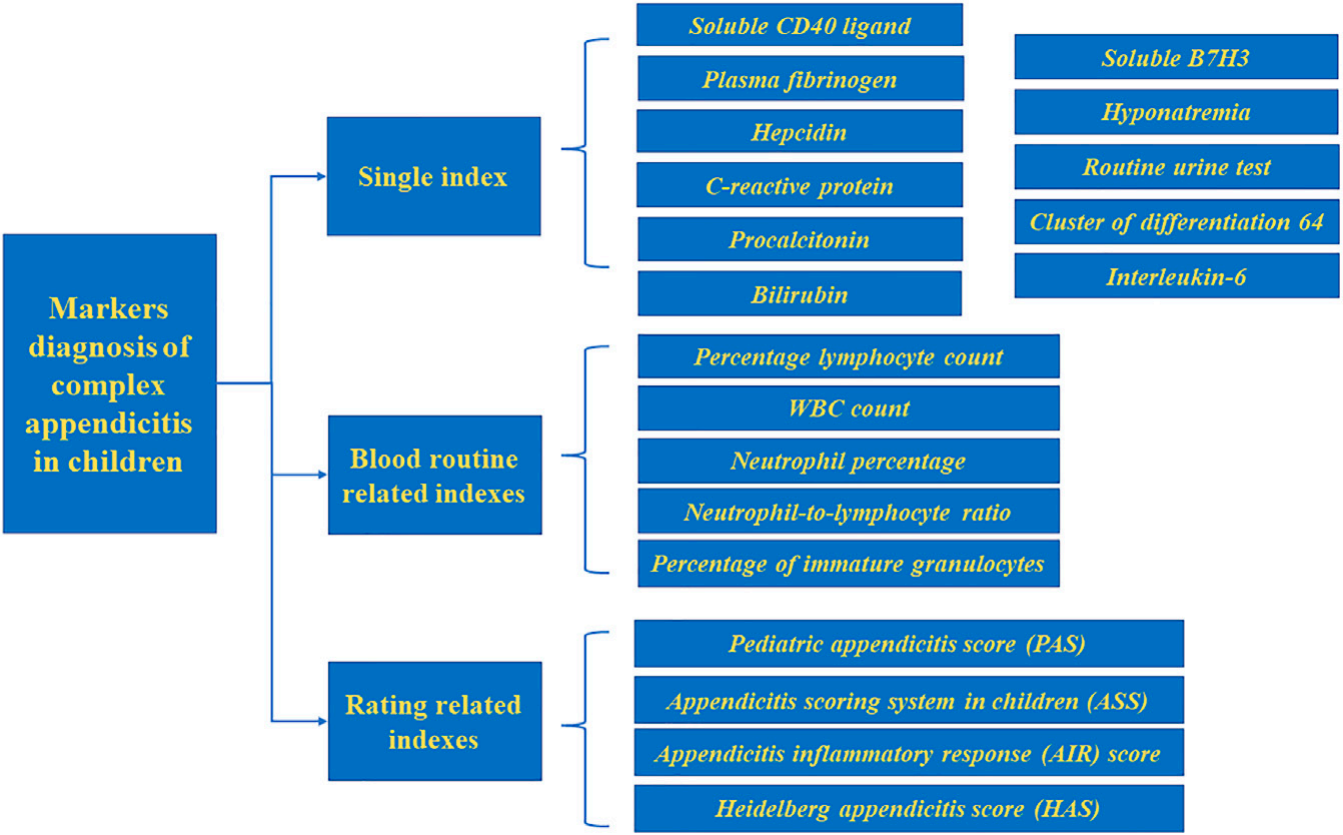
Markers for pediatric complex appendicitis.

Acute appendicitis can be divided into simple appendicitis (SA) and complex appendicitis (CA) according to the severity of the disease. Diagnosis of CA is based on appendix perforation, appendix gangrene, appendix abscess, intra-abdominal abscess, and fecal peritonitis (Pham et al., 2016; Hajibandeh et al., 2020), CA is more common in children, with a prevalence of up to 30% (Yu et al., 2019). However, due to nonspecific symptoms and difficulties in accurate physical examination, distinguishing between SA and CA in children remains a challenge. The application of biomarkers in the diagnosis of CA has the advantages of easy collection, no limitations based on operator skill, and no radiation exposure compared with other diagnostic modalities. Our study summarizes biomarkers and the scoring system related to the diagnosis of CA in children to diagnose this disease more quickly and increasing the time for follow-up treatment. (Figure 1).

## Single Index

### Soluble CD40 Ligand

sCD40L has both pro-thrombotic and pro-inflammatory effects (de Lizarrondo et al., 2012; Seibold and Ehrenschwender, 2015; Liu et al., 2018). When the body experiences an inflammatory response, sCD40L stored in unstimulated platelets aggregates, followed by stimulation of nuclear factor-κB signaling, causing upregulation of pro-inflammatory and pro-thrombotic factors.

Studies have shown that sCD40L has excellent sensitivity and specificity in predicting CA in children. sCD40L levels below 178.00 pg/ml in the first 3 days of appendicitis can exclude the diagnosis of ruptured appendicitis (RA). Conversely, sCD40L above 301.00 pg/ml confirms the diagnosis of appendicitis and may have a high probability of RA (Huang et al., 2021). In these cases, further testing may not be necessary to confirm the diagnosis in patients with suspected appendicitis.

### Plasma Fibrinogen

Under physiological conditions, the plasma concentration of fibrinogen ranges from 2 to 4 g/L (Tennent et al., 2007). However, under pathological conditions, such as infection, post-injury inflammation or diseases associated with vascular rupture, plasma fibrinogen concentrations can increase several-fold (Luyendyk et al., 2019). Therefore, fibrinogen is considered to be a marker of acute inflammation (Adams et al., 2004). An increase in fibrinogen in the blood can indicate that inflammation has been increased in the organism; it can also indicate the development of vascular inflammatory disease (Kayapinar et al., 2019; Luyendyk et al., 2019).

Studies have shown that fibrinogen has relatively high specificity and acceptable sensitivity as a laboratory marker for predicting perforated appendicitis (PA) (Feng et al., 2014). Fibrinogen can also be an important indicator to exclude CA (Li et al., 2011). Children with plasma fibrinogen levels > 520 mg/dl are more likely to have CA (Prada-Arias et al., 2017).

### Hepcidin

Hepcidin is synthesized in hepatocytes and is a major hormonal regulator of iron metabolism, an antimicrobial peptide, and an acute phase reactant. For healthy children, the interquartile range for hepcidin was shown to be 21.90 ng/ml (Kumar et al., 2019). In inflammatory and infectious conditions, hepcidin synthesis is regulated by interleukin (IL)-6 and lipopolysaccharide (Rodriguez et al., 2014; Arezes et al., 2015). Hepcidin has direct antimicrobial activity and helps host defense by depriving microorganisms of this essential iron mineral (Ganz, 2003; Michels et al., 2015).

Kaiser et al. found that the serum hepcidin level of SA and CA in children was significantly increased. In addition, the accuracy of the combination of leukocytes and C-reactive protein (CRP) for the diagnosis of acute appendicitis can be improved by increasing serum hepcidin levels (Kaiser et al., 2018).

### CRP

CRP is an acute temporal protein important for detecting occult inflammation and active disease (Clyne and Olshaker, 1999). Many researchers believe CRP has good diagnostic value for CA. Perforation should be considered in children with high CRP levels and free fluid or abscess formation by ultrasonography (Boettcher et al., 2017). Yang et al. found that increased WBC levels, CRP levels, and absolute value of neutrophils were associated with an increased likelihood of perforation (Yang et al., 2019). Beltran et al. (Beltrán et al., 2007) indicated that the CRP level and its sensitivity increased gradually from symptom to diagnosis, and the specificity at 12, 24, and 48 h from symptom to diagnosis was still very high (90%). However, the diagnostic accuracy of CRP reached its highest value within 12 h, after which it decreased significantly.

Different researchers have reached different conclusions regarding the threshold value of CRP for diagnosis of CA. Several studies have shown that children with CRP values in the range of 10–50 mg/L suggest uncomplicated appendicitis, while CRP > 50 mg/L strongly suggests CA (Kafetzis et al., 2005; Xharra et al., 2012). It has also been suggested that CRP values > 50 mg/L are more likely to indicate CA (Wu et al., 2012). A retrospective study by Zani et al. found that CRP and WBC levels increased in proportion to the severity of appendicitis. Children with CRP below 40 mg/L had an 80% chance of not having CA (Zani et al., 2017).

### Procalcitonin

PCT is a good marker of severe bacterial infection. PCT levels increased with the severity of infection (Meisner, 2014). PCT is less accurate than CRP and WBC in the diagnosis of acute appendicitis, but more accurate in the diagnosis of CA (Yu et al., 2013; Cui et al., 2019). Patients with PCT levels > 0.18 ng/ml and/or CRP > 3 mg/dl are at higher risk of peritonitis and should be closely monitored; more stringent treatment should be administered early (Gavela et al., 2012).

### Bilirubin

Hyperbilirubinemia is defined as bilirubin levels greater than 20.5 μmol/l (Emmanuel et al., 2011). One prospective study showed that an increase in total serum bilirubin can be used as an indicator of appendicitis perforation in children (Pogorelić et al., 2021a). Bilirubin levels are highly specific for the diagnosis of complicated appendicitis; a 2.0-fold increase in the likelihood of complicated appendicitis was observed in patients with elevated bilirubin levels (Noh et al., 2012). In addition, total bilirubin > 21.38 mol/L was a predictor of appendicitis perforation (Yamazaki et al., 2021). As serum bilirubin level is an economical, simple, and available laboratory index, it should be recommended for preliminary evaluation of acute appendicitis in pediatric patients.

### Soluble B7H3

B7H3, an immune checkpoint molecule belonging to the B7-CD28 family, is associated with the regulation of T cells (Janakiram et al., 2017). Release of sB7H3 may regulate B7H3R/B7H3 interactions *in vivo* (Zhang et al., 2008). This marker has been increasingly used to detect a number of inflammatory conditions (Chen et al., 2009; Chen et al., 2013a; Xu et al., 2019). Du et al. found that sB7H3 is important in predicting acute appendicitis and its severity in children, and sB7H3 > 36.146 ng/ml is statistically significant for the diagnosis of CA. The combination of CRP and sB7H3 increases the accuracy of PA diagnosis (Du et al., 2020).

### Hyponatremia

Hyponatremia refers to a serum sodium concentration ≤ 135 mmol/L. Research suggests hyponatremia may be a useful tool for predicting PA (Pogorelić et al., 2021b). It is unclear why hyponatremia usually accompanies CA patients, but it may be mediated by antidiuretic hormone (Käser et al., 2013; Pham et al., 2016; Pogorelić et al., 2019; Yang et al., 2019; Giannis et al., 2020; Lindestam et al., 2020).

### Routine Urine Test

A routine urine test is helpful to distinguish SA from PA. Chen et al. (Chen et al., 2013b) found that urinary ketone bodies, nitrate, urinary specific gravity, pH, WBC count and red blood cell (RBC) count all appeared to be important predictors of PA. Compared with children with SA, children with PA are more likely to be positive for ketone bodies and nitrates, higher urinary proportion, lower urinary pH, more urinary WBCs, and more urinary RBCs. In addition, urine RBC count (≥ 2.0/hpf) and WBC count (≥ 4.0/hpf) can be important predictors of appendiceal perforation or appendiceal abscess in children.

### Cluster of Differentiation 64

Quantitative expression of neutrophil CD64 serves as a sensitive and specific laboratory indicator of the presence of sepsis or systemic acute inflammatory response, thus suggestive of a variety of inflammatory conditions (Xini et al., 2019; Hashem et al., 2020; Patnaik et al., 2020). Levels of CD64 were found to predict the occurrence of advanced appendicitis or PA; CRP levels and CD64 expression on leukocytes could better predict the diagnosis of CA (Ozguner et al., 2014).

### Interleukin (IL)-6

IL-6 is an important natural immune cytokine closely related to the degree of inflammation. Researchers often use IL-6 as an indicator of the degree of systemic inflammation (Raeburn et al., 2002). IL-6 plays an important role in differentiating simple and advanced cases of appendicitis (Türkyilmaz et al., 2006).

## Blood Routine Related Indexes

### Percentage Lymphocyte Count

Virmani et al. demonstrated that the percentage lymphocyte count is a better indicator than the neutrophil to lymphocyte ration (NLR) and total leukocyte count (TLC) in distinguishing SA from CA. The threshold value for lymphocyte count is 14.8%. Values less than this are considered CA whereas values greater are considered SA (Virmani et al., 2018; Celik et al., 2019).

### WBC Count

WBC count is not sensitive and specific enough to distinguish PA from non-perforated appendicitis (Grönroos, 2001). However, the use of CRP alone or WBC count in combination with CRP helps to differentiate between PA and non-perforated appendicitis (Grönroos, 2001). CRP levels > 50 mg/l and leukocyte counts > 104/mm^3^ were effective adjuncts to predict appendiceal perforation (Kafetzis et al., 2005; Buyukbese Sarsu and Sarac, 2016; Yang et al., 2019; Zvizdic et al., 2021).

However, some studies have reached an opposite conclusion, suggesting the increase in leukocyte count is a risk factor for CA (Beltrán et al., 2007; Siddique et al., 2011; Şahbaz et al., 2014), and its sensitivity increases with the duration of symptoms (Beltrán et al., 2007; Ngim et al., 2014). Okamoto et al. (Okamoto et al., 2006) found that an increase in WBC count 48 h after the onset of pain is a prognosis marker of CA. In addition, Beltra et al. (Beltrán et al., 2007) demonstrated that WBC count can also distinguish between SA and PA. The diagnostic accuracy was high (80%) at 12 and 48 h, and beyond 49 h, decreasing to 70% at 24 h.

### Neutrophil Percentage

Neutrophil percentage can be used to diagnose CA, with elevated neutrophil percentage (> 74%) and CRP (> 8 mg/dl) levels predicting a more than five-fold increased risk of PA (Yang et al., 2019). A neutrophil count greater than 75% is considered CA (Virmani et al., 2018).

### Neutrophil-to-Lymphocyte Ratio

Neutrophil-to-lymphocyte ratio (NLR) is a simple and easily calculated marker of the body’s inflammatory status (Käser et al., 2010). Because it provides information about two different inflammatory and immune pathways, we believe NLR is valuable in predicting appendicitis and its severity. Hajibandeh et al. demonstrated that children with NLR > 8.8 are at higher risk of CA (Hajibandeh et al., 2020).

### Percentage of Immature Granulocytes

In recent years, it has been found that the Ig percentage can be used as a marker of infection and that this percentage can be measured automatically in a new generation of hemograms. It has the advantage that it can be measured easily and quickly without incurring additional costs (van der Geest et al., 2014; Pavare et al., 2018; Zeng et al., 2020). Studies have demonstrated that an elevated Ig percentage can predict CA, with a sensitivity of 85.4% and a specificity of 61.5% when the Ig percentage is 35%. Because it is quick and easy to measure, does not require additional blood collection, and does not incur additional costs, IG percentage may be the test of choice for diagnosing patients with CA (Güngör et al., 2021). (Table 1)

**TABLE 1:** Related markers in the diagnosis of complex appendicitis in children and corresponding values.

marker	Value
sCD40L	>301.00 pg/ml
Plasma Fibrinogen	>520 mg/dl
CRP	>50 mg/L/>30 mg/L
PCT	>0.18 ng/m
Bilirubin	>21.38 mol/L
sB7H3	>36.146 ng/ml
Serum natrium	≤135 mmol/L
Urine RBC counts	≥2.0/hpf
Urine WBC counts	≥4.0/hpf
Percentage lymphocyte count	<14.8%
WBC	>13,500/mm^3^
Percentage neutrophil counts	>74%
NLR	>8.8
IG%	>35%

CRP, C-reactive protein; PCT, procalcitonin; RBC, red blood cell; WBC, white blood cell; NLR, neutrophil-to-lymphocyte ratio; IG, immature granulocyte.

## Rating Related Indexes

### Pediatric Appendicitis Score

PAS includes the following indicators: 1) cough/shock/jumping abdominal pressure in the right lower abdomen, 2), anorexia, 3), fever, 4), nausea/vomiting, 5), pain in the right iliac fossa, 6), leukocytosis, 7), polymorphonuclear neutrophilia, and 8) painful migration. All of these variables were scored as 1 except for signs (1 and 5), which were scored as 2, for a total score of 10. The score is now widely used to diagnose acute appendicitis in children. A score ≥ 6 is consistent with a diagnosis of appendicitis (Samuel, 2002). PAS may be related to the pathological progression of appendicitis and the severity of the disease. PAS ≥ 8 can be used for the diagnosis of CA (Fujii et al., 2020). Fujii et al. demonstrated that symptom duration > 1 day, CRP > 4 mg/dl and PAS ≥ 8 predicted CA, which was more convincing than a single indicator of any of these three.

### Appendicitis Scoring System in Children

Lee et al. developed a scoring system capable of differentiating CA in children under 10 years of age, which consisted of five risk factors: diarrhea, anorexia, temperature, CRP level, and presence of periappendiceal free fluid on radiological examination. Among them, fever (Bonadio et al., 2018; Obinwa et al., 2015; Peng et al., 2006; van den Bogaard et al., 2016; Atema et al., 2015; Augustin et al., 2011) and CRP level (van den Bogaard et al., 2016; Lee et al., 2021; Barreto et al., 2010; Bröker et al., 2012) were found to be predictors of CA in previous studies. The advantages of this score over other scores is that it includes CRP levels and excludes indistinguishable symptoms, such as pain metastasis and nausea.

To reduce the risk of delaying treatment due to misclassification of CA as uncomplicated appendicitis using this scoring system, this score uses a score of 4 as the threshold value to distinguish CA from SA. Appendectomy should be considered if the patient meets both an ASS score of four and CRP ≥ 50 mg/L or has two or more risk factors (Lee et al., 2021).

### Appendicitis Inflammatory Response Score

The AIR score includes vomiting, right iliac fossa pain, muscle tension, temperature, neutrophil grading, WBC, and CRP. Because it is primarily based on objective inflammatory markers, this score has the advantage of high repeatability in different environments, independent of the inspector’s experience. Pogoreli et al. found that the AIR score was able to distinguish PA from non-perforated appendicitis; ≥ 9 (AIR score) is a good index of appendix perforation, with a sensitivity of 89.5% and a specificity of 71.9% (Pogorelić et al., 2021c).

### Heidelberg Appendicitis Score

HAS includes four factors (persistent pain, right lower abdominal tenderness, rebound tenderness, and appendicitis by ultrasound). Current studies have shown that perforation in children with appendicitis can be identified by using HAS as it can reliably detect PA in children and exclude perforation if the score is negative (Boettcher et al., 2017).

Stiel et al. proposed a modified Heidelberg score including ultrasound showing appendicitis, CRP > 20 mg/L, rebound tenderness, leukocytes > 11 × 10^9^/L and right lower abdominal tenderness. Modified Heidelberg appendicitis provides good predictability for both general appendicitis and PA (Stiel et al., 2020). (Table 2)

**TABLE 2:** Different scoring systems for diagnosing complex appendicitis in children, including the different weighing factors for each score.

	PAS	ASS	AIR	HAS	Mod HAS
Nausea/Vomiting	1		1		
Pain in RIF	2		1	1	1
Abdominal Defense (Low/Mild/Severe)			1/2/3		
Temperature >38.5°C	1	3	1		
Neutrophilia (70–84%/>85%)	1		1/2		
Leukocytes (10.0–14.9 × 109/l/>15.0 × 109/l/>11 × 109/l)	1		1/2		1
CRP (10–49 g/l/>50 g/l/>20 mg/l)		5	1/2		1
US demonstrating APP				1	1
Rebound tenderness				1	1
Anorexia	1	2			
Diarrhea		2			
Periappendiceal free fluid on image		3			
Continuous pain				1	
Cough/Percussion/Hopping tenderness	2				
Migration of pain	1				
Positive score	8/10	4/15	9/12	3/4	3/5

RIF, right Iliac Fossa; CRP, C-reactive protein; US demonstrating appendicitis comprises the appendix diameter > 6 mm and/or signs of inflammation such as wall edema, hyperemia, and surrounding inflammation; US, ultrasound; APP, appendicitis.

## Conclusion

Research on relevant markers for the diagnosis of CA in children is gradually increasing. Biomarkers and scoring systems for children allow for earlier diagnosis, which not only reduces the number of unnecessary surgeries, but also reduces complications and helps to significantly reduce the cost of treating patients with acute abdominal disease. All markers list in the manuscript are helpful for diagnosis of CA, however, no index can diagnose CA at an accuracy of 100%; based on the overall consideration, we recommend PAS. Although, the review focuses the markers for the precision diagnosis of complex appendicitis in children. related markers for CA are as same as medical history, physical examination and imaging examinations. Appropriate selection of diagnostic markers and scoring systems for predicting CA in children is important for determining the best treatment strategy. There are several non-routine indexes for diagnosis of CA; therefore, more researches about the non-routine indexes need to be performed to verify their significance and they can be routine test for diagnosis of CA. The research on some biomarkers is still in its infancy, and further investigation is needed to refine the reference value for diagnosing CA, diagnostic accuracy, and clinical applications of CA.
